# Advances in prostate cancer imaging

**DOI:** 10.12688/f1000research.14498.1

**Published:** 2018-08-24

**Authors:** Matthew R. Tangel, Ardeshir R. Rastinehad

**Affiliations:** 1 Department of Radiology, Mount Sinai Health System, New York, NY, 10003, USA; 2 Department of Interventional Radiology, Mount Sinai Health System, New York, NY, 10003, USA

**Keywords:** Multiparametric MRI, PSMA-PET/CT, Ultrasound elastography, Prostate cancer

## Abstract

Many exciting advances in medical imaging have been made in recent years that will alter the way we diagnose, stage, and treat patients with prostate cancer. Multiparametric magnetic resonance imaging (MRI) is emerging as the main modality for prostate cancer imaging. Contrast-enhanced ultrasound and shear wave elastography may be strong alternatives in patients who cannot undergo MRI. Prostate-specific membrane antigen-directed positron emission tomography/computed tomography has proven to be valuable in the primary staging of high-risk disease and for detecting disease in patients with biochemical recurrence. As more studies continue to emerge, it is becoming clear that the standard algorithm for diagnosing and staging prostate cancer will undergo significant changes in the near future.

## Introduction

Over the past several years, many advances have been made in the field of advanced imaging in the diagnosis of prostate cancer that will likely have strong sustained effects. The results of recent studies, such as those of the prostate magnetic resonance imaging (MRI) study (PROMIS), are already being applied in clinical practice. Multiparametric MRI (mpMRI) is one example of an imaging modality that has recently been implemented to aid the diagnosis and treatment of prostate cancer. Additional encouraging innovations include the use of contrast-enhanced ultrasound, ultrasound elastography, and prostate-specific membrane antigen-directed positron emission tomography (PSMA-PET). In particular, PSMA-PET shows much promise for the identification of local tumor recurrence in patients with a rising prostate-specific antigen (PSA) status post radical prostatectomy in whom conventional imaging would otherwise be of limited sensitivity. The objective of this article is to give a brief review on upcoming advances in prostate cancer imaging and provide an overview of what may be more widely available for clinical practice in the near future.

## Prostate magnetic resonance imaging

Currently, men who are identified to be at risk of prostate cancer undergo a transrectal ultrasound (TRUS)-guided biopsy. Unfortunately, it is known that TRUS has limited sensitivity and specificity for the detection of prostate cancer ^1^. Therefore, biopsies taken via this method are done somewhat “blindly” throughout the prostate, which can result in a high false-negative rate, misrepresentation of the true tumor burden, and the potential for complications including urinary tract infection, Gram-negative bacteremia, dysuria, and pain/discomfort ^2– 4^. In contrast, most other solid organ cancers are first identified by imaging and then image-guided biopsies are obtained to increase the yield. PROMIS sought to utilize imaging alone via mpMRI in all men with suspected prostate cancer to bring the screening of prostate cancer up to the current standards of other organ systems. It found that the sensitivity of mpMRI for clinically significant cancer is around 93%, with a negative predictive value of 89%. The specificity was 41% with a positive predictive value of 51%, meaning mpMRI is more sensitive than TRUS-guided biopsy (93% versus 48%), but TRUS-guided biopsy had better specificity (41% versus 96%) ^5^. PROMIS suggests that if mpMRI were used as a triage test, approximately one in four men may be able to safely avoid prostate biopsy owing to its high negative predictive value (89% for mpMRI and 74% for TRUS). The limitation of mpMRI is its low specificity, meaning that an image-guided biopsy would still be required in men with suspicious mpMRI findings ^5^. However, this may be subject to change in the future with the advent of more modern technology and newer methods of prostate imaging. For example, PROMIS used T1W, T2W, DWI, and dynamic gadolinium-enhanced imaging with a 1.5 Tesla scanner and pelvic phase array. Currently, 3 Tesla scanners are becoming more commonplace, and endorectal coils are now used to increase the signal-to-noise ratio and thus image quality; both demonstrate potential to increase sensitivity for the detection of prostate cancer ^6, 7^. One recent study that included these technologies has already demonstrated that MRI before biopsy and MRI-targeted biopsy is superior to TRUS-guided biopsy in biopsy-naïve men deemed at risk for prostate cancer ^8^.

One alternative to mpMRI as a triage test is to perform mpMRI in patients who underwent a negative prostate biopsy and had some indication for repeat biopsy or who tested positive and required reclassification. The prostate imaging compared to transperineal ultrasound-guided biopsy for significant prostate cancer risk evaluation (PICTURE) trial sought to use mpMRI as a means of risk stratifying men with a negative TRUS-guided biopsy to determine whether a repeat TRUS-guided biopsy would be necessary or mpMRI could be obtained instead. The results demonstrate that in men who may require repeat biopsies, mpMRI can safely rule out clinically significant prostate cancer because of its high sensitivity and negative predictive value. Specifically, men with low-risk findings on mpMRI have a 10% chance of clinically significant cancer ^9^. Initially, widespread mpMRI use was not believed to be feasible owing to concerns over cost-effective care ^10^. However, recent studies indicate that mpMRI is a viable approach for the early detection of prostate cancer from a cost-effectiveness perspective not only in biopsy-naïve patients but also in patients with previous negative biopsies as well ^11^. Nonetheless, mpMRI will likely be used in the future for risk stratifying patients who would have otherwise undergone repeat TRUS-guided biopsies.

## Multiparametric ultrasound

Historically, TRUS-guided biopsy was and continues to be used for detecting suspected sites of neoplastic proliferation as well as local staging ^12^. The standard technique that is utilized is grayscale ultrasonography. As alluded to above, grayscale is not accurate at identifying a large percentage of prostate cancers. Approximately 50% of prostate cancers appear hypoechoic, but as many as 30% are isoechoic to the surrounding normal tissue and thus not easily identifiable ^13^. In addition, only one-third of hypoechoic lesions end up being prostate cancer, rendering only about 40% of prostate cancers detectable on grayscale ultrasonography ^13, 14^. In an attempt to circumvent the shortcomings of standard grayscale imaging, multiple other ultrasound techniques have been applied.

Color and power Doppler ultrasound may play some role in detecting prostate cancer. The general principle is that prostate cancer requires angiogenesis in order to develop into clinically significant disease ^15^. Both color and power Doppler can help identify hyperemic tissue and therefore potential prostate cancer. Power Doppler is more sensitive than color Doppler, but neither of them is sensitive enough to detect early stage prostate cancers. The limited resolution results in the detection of vessels in the millimeter range, whereas angiogenesis can result in vessels as small as 10–50 micrometers ^15^. Therefore, the utility of color and power Doppler ultrasonography may be restricted to detecting only higher Gleason grade tumors ^16^.

Contrast-enhanced ultrasound (CEUS) is a relatively new technique that is not frequently used in the United States in the clinical setting. It involves the intravenous injection of gas-filled microbubbles that are comparable to the size of erythrocytes just prior to or during ultrasound image acquisition ^17^. The injected microbubbles enhance the backscatter of ultrasound waves, resulting in the amplification of signals from blood flow. The ultrasound transducer recognizes these regions and creates images from both the nonlinear oscillation of microbubbles and the microbubble destruction ^18^. Prostate cancer, even in its early stages, generally has increased flow due to angiogenesis and on CEUS will demonstrate asymmetric rapid inflow, increased focal enhancement, and asymmetry of intraprostatic vessels that are beyond the resolution of conventional techniques including color and power Doppler ^19^. An algorithm is used to calculate a peak intensity value, which corresponds to the degree of enhancement; one study concluded that this can help differentiate between benign and malignant lesions ^20^.

One other promising area in the field of sonography is the use of ultrasound elastography, more specifically shear wave elastography (SWE). SWE is currently being utilized for staging chronic liver disease and has numerous other potential beneficial applications as well ^21^. SWE, in contrast to strain elastography (SE), is dependent solely on Young’s modulus and the pulse transmitted by the ultrasound transducer; therefore, there is less variability compared to SE, which relies on transducer compression, resulting in significant interobserver variability ^22^. The stiffness of prostate cancer relative to normal prostatic tissue is known, and using SWE can help distinguish neoplastic lesions ^23^. A recent systematic review of SWE alluded to sensitivity and specificity values comparable to those seen in mpMRI ^24^. This implies that SWE may be appropriate independently for elucidating targets during ultrasound-guided biopsies.

## Prostate-specific membrane antigen-directed positron emission tomography/computed tomography

PSMA is expressed on the cell surface of normal prostate tissue and is overexpressed in prostate cancer by several orders of magnitude. Therefore, this antigen was used as a target for prostate cancer-specific imaging ^25^. Initially, PSMA was tagged with Indium 111, but the lack of internalization by viable prostate epithelial cells led to limited use in diagnosing tumors within the prostate gland and seminal vesicles ^26^. ^68^Gallium-labeled PSMA, on the other hand, demonstrates high tumor-to-background contrast and is a small molecule, which aids in internalization and the detection of primary prostate tumors ^27^. The highest perceived benefit of ^68^Gallium-PSMA ligand PET/CT is in the primary staging of high-risk disease and for detecting disease in patients with biochemical recurrence. Preliminary data also show that ^68^Gallium-PSMA ligand PET/CT may be beneficial for biopsy targeting after a previous negative biopsy in a patient highly suspected of having prostate cancer, especially when combined with mpMRI ^28^. Currently, a multicenter prospective randomized controlled trial is taking place in Australia that aims to compare the diagnostic accuracy of PSMA-PET/CT to conventional imaging in detecting nodal and distant metastatic disease ^29^. One study has already shown that PSMA-PET may be complementary to stand-alone mpMRI for tumor localization (sensitivity of PET 64%, mpMRI 58%, and ^68^Ga-PSMA-PET/MRI 76%) ^30^. The National Comprehensive Cancer Network (NCCN) states that PET/CT can be considered in patients with PSA persistence/recurrence status post radical prostatectomy utilizing both C-11 choline or F-18 fluciclovine, a relatively new addition ^31^.

## Conclusion

Emerging technology is changing how we approach the diagnosis of prostate cancer from an imaging standpoint. mpMRI continues to demonstrate utility in risk stratifying patients with suspected prostate cancer and will be the foundation for prostate cancer imaging in the future. SWE may independently be used to identify suspected cancer during TRUS-guided biopsies. The emergence of PET in the diagnostic work-up of prostate cancer, specifically PSMA-PET, shows promise in the detection of recurrence in patients where conventional imaging (whole body bone scan, CT) may be negative. Overall, new imaging technology will allow for more accurate diagnosis, staging, and treatment follow-up in patients with prostate cancer in the near future.
